# Two new species of the genus *Miasa* Distant, 1906 from China, with a key to all species (Hemiptera, Fulgoromorpha, Dictyopharidae)

**DOI:** 10.3897/zookeys.754.23525

**Published:** 2018-04-30

**Authors:** Yan-Li Zheng, Lin Yang, Xiang-Sheng Chen, Xu-Qiang Luo

**Affiliations:** 1 Institute of Entomology, Guizhou University; The Provincial Special Key Laboratory for Development and Utilization of Insect Resources of Guizhou, Guizhou University, Guiyang, Guizhou, 550025 P. R. China; 2 School of Geography and Tourism, Guizhou Education University, Guiyang, Guizhou, 550018 P. R. China; 3 Institute of Environmental, Resources and Disaster, Guizhou Education University, Guiyang, Guizhou, 550018 P. R.China

**Keywords:** Fulgoroidea, Oriental region, planthopper, taxonomyt. Text.

## Abstract

Two new species *Miasa dichotoma* Zheng & Chen, **sp. n.** and *M. trifoliusa* Zheng & Chen, **sp. n.** from China are described and illustrated. A key of identification to all species of the genus is provided.

## Introduction

The Oriental genus *Miasa* was established by Distant (1906) for a single species *Elidiptera smaragdilinea* Walker, 1857, from Malacca (Malay Peninsula). Song et al. (2014) reviewed this genus revising the already three known species and adding two new. In this paper, two new species, *M. dichotoma* sp. n. and *M. trifoliusa* sp. n. are described and illustrated, with photographs of the adult habitus. So far, this genus now includes seven species.

## Materials and methods

The morphological terminology and measurements follow Yang and Yeh (1994) and Song et al. (2014). Specimens are deposited in the Institute of Entomology, Guizhou University, Guiyang, China (IEGU). Dry specimens were used for the observation, description, and illustration. Genital segments of the examined specimens were macerated in boiling solution of 10% potassium and drawn from preparations in glycerine jelly under a Leica MZ12.5 stereomicroscope. Color pictures for adult habitus were obtained by a KEYENCE VHX-1000 system. Illustrations were scanned with Canon Cano Scan LiDE 200 and imported into Adobe Photoshop CS6 for labelling and plate composition. The type specimens are deposited in the Institute of Entomology, Guizhou University, China (IEGU).

The following abbreviations are used in the text:

**BL** body length (from apex of cephalic process to tip of fore wings);

**HL** head length (from apex of cephalic process to base of eyes);

**HW** head width (including eyes);

**FWL** forewing length.

## Taxonomy

### Miasa

Taxon classificationAnimaliaHemipteraDictyopharidae

Distant, 1906

Figs 1–15 , 16–20 , 21–32

Miasa Distant, 1906: 247; Schmidt 1906: 280; Melichar 1912: 37; Schmidt 1915: 348; Distant 1916: 28; Schmidt 1928: 129; Metcalf 1946: 34; Song et al. 2014: 142. Type species. Elidiptera smaragdilinea Walker, 1857, by original designation.
Putalamorpha Bierman, 1910: 9. Type species. Stenocranus productus Lethierry, 1888 by original designation. Synonymised by Melichar 1912: 79.

#### Type species.

*Elidiptera smaragdilinea* Walker, 1857.

#### Diagnosis.

For the relationships and diagnosis of *Miasa* see Songet al. (2014).

#### Distribution.

Burma; Indonesia (Borneo, Jawa, Sumatra); Malaysia (Borneo, Sabah, Sarawak, peninsula); China (Yunnan); Thailand; Vietnam; Singapore; Myanmar (ex Burma).

#### Key to the species of the genus *Miasa* based on males

(Modified from Songet al. 2014 and updated two new species)

**Table:** 

1	Frons below eyes including median carina uniformly dull ochreous; pronotum with posterolateral corner pale yellow to ochreous with or without dark spot behind eye; forewings posterior margin broadly dull ochreous; aedeagus with two pairs of ventral lobes and a pair of dorsolateral lobes	**2**
–	Frons below eyes emerald green, with median carina testaceous or if uniformly dull ochreous medial carina darker; pronotum with lateroventral corner brown, without dark spot behind eye; forewings with inner margin of clavus narrowly dark brown; aedeagus with two pairs of ventral lobes, but without dorsolateral lobes; southern Malay Peninsula, Sumatra and Java	***M. smaragdilinea* (Walker)**
2	Male segment X broad basally, not hatchet-shaped in lateral view	**5**
–	Male segment X narrow basally, hatchet-shaped in lateral view	**3**
3	Aedeagus with membranous phallobase bearing two pair of lobes, a pair of dorsal, a pair of complicated ventral lateral lobes (Fig. 14); southwestern China	***M. dichotoma* Zheng & Chen, sp. n.**
–	Aedeagus with membranous phallobase bearing three pairs lobes (Fig. 31)	4
4	Phallobase membranous with a pair of dorsal lobes directed posteriorly, and two pairs of ventral lobes: upper pair large and elongate, directed dorsally; lower pair relatively small and rounded; southwestern China, southeast Asia to North Malay Peninsula	***M. wallacei* Muir**
–	Phallobase membranous with a pair of dorsal lobes directed posteriorly, and two pairs of ventral lobes (Fig. 31): large, almost equal length, apical bifurcate, directed dorsally; southwestern China	***M. trifoliusa* Zheng & Chen, sp. n.**
5	Preocular field with a blackish brown spot; male segment X with ventral margin weakly incurved in lateral view	**6**
–	Preocular field without blackish brown spot; male segment X with ventral margin distinctly incurved sub-basally in lateral view; Borneo	***M. borneensis* Song, Webb & Liang**
6	Upper process of gonostyle distinctly broad at apex; basal ventral lobes of aedeagus distinctly short and small; male segment X with apical ventral margin distinctly produced in a long process in lateral view; Borneo	***M. nigromaculata* Song, Webb & Liang**
–	Upper process of gonostyle not broad at apex; basal ventral lobes of aedeagus distinctly long; male segment X with ventral margin not protruded in lateral view; Sumatra and Java	***M. producta* (Lethierry)**

### Miasa dichotoma

Taxon classificationAnimaliaHemipteraDictyopharidae

Zheng & Chen sp. n.

http://zoobank.org/792EE97D-AA25-4C91-8A93-B771A0B70699

Figs 1–15 , 16–20

#### Measurements.

♂, BL: 14.8–15.1 mm; HL: 2.0–2.3 mm; HW: 0.8–0.9 mm; FWL: 11.1–11.3 mm. ♀, BL: 15.6 mm; HL: 2.6 mm; HW: 1.0 mm; FWL: 11.5 mm.

#### Description.

General colour in dried specimens ferruginous-brown, marked with pale green and black. Cephalic process of the base brown, terminal black, brown on side. Frons uniformity brown. Frontoclypeal area dark with brown freckles. Compound eyes dark brown; ocelli light pink. Antennae brown. Pronotum and mesonotum brown, the median area emerald green. Forewings with stigmal area and posterior margin broadly dull ochreous, a large oblique triangular apical streak, and a narrow streak along nodal line fuscous; hind wings with an apical fuscous spot. Legs brown with dark spots.

**Figures 1–15.**
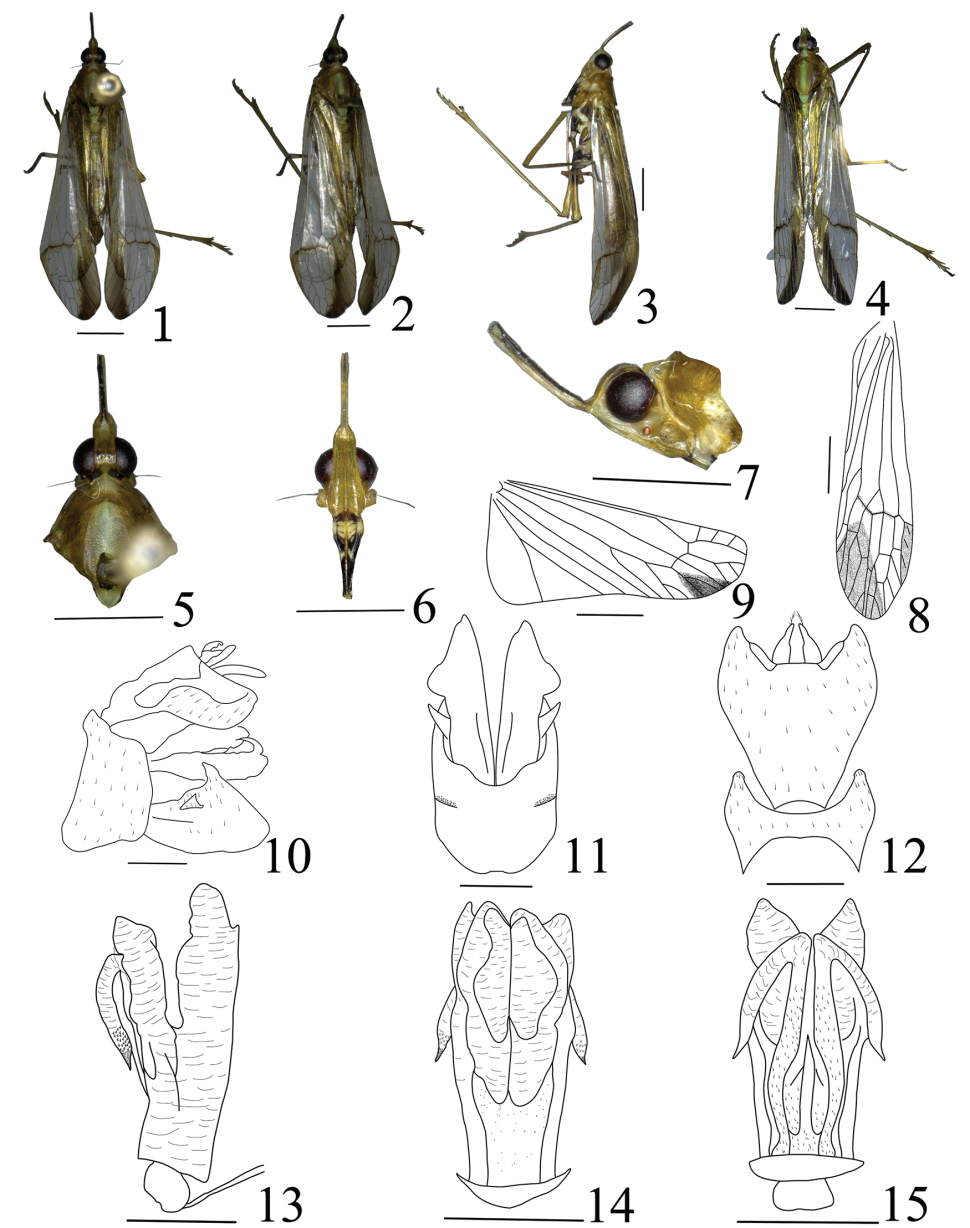
*Miasa dichotoma* sp. n.: **1** male, holotype, dorsal view **2** male, oblique side view **3** male, lateral view **4** female, dorsal view, spoiled **5** male, head and thorax, dorsal view **6** male, frons and clypeus, ventral view **7** male, head and pronotum, lateral view **8** male, forewing **9** male, hind wing **10** genitalia, lateral view **11** pygofer and parameres, ventral view **12** pygofer and anal tube, dorsal view **13** aedeagus, lateral view **14** aedeagus, ventral view **15** aedeagus, dorsal view. Scale bars: 2 mm (**1–9**), 0.5 mm (**10–15**).

Cephalic (Figs 1, 2, 5–7) process relatively long, distinctly upturned, ratio length to length of pronotum and mesonotum combined 0.8. Vertex (Figs 1, 2, 5–7) with lateral margins carinate, sub-parallel at base, sharply sinuate in front of eyes, then narrowing to arrowhead at apex, ratio of length to width between eyes 4.5. Frons (Fig. 6) elongate, median carina complete and elevated, length approx. 3.9 times long than width. Pronotum (Figs 1, 2, 5–7) distinctly shorter than mesonotum medially in the middle line, median carina obscure, lateral carina distinct, ratio length to length approx. 0.3:1. Mesonotum (Figs 1, 2, 5–7) median carina obscure, lateral carina distinct. Forewings (Figs 1, 8) elongate, with ratio of length to width approx. 4.0:1; CuA vein first branched before Sc+R and M veins near middle; crossveins very scarce, forming a nodal line along Sc+R, M and CuA veins at apical 1/3; apical cells approx. 10–12; Pcu and A_1_ veins fused into a long Pcu+A_1_ vein at apical 1/6 in clavus; stigmal area clear, with four cells. Legs long and thin, profemur not flattened and dilated, with one minute, short, blunt spine near apex; metatibia with 6 lateral black-tipped spines and 6 apical black-tipped teeth, hind tibiae I with nine and tarsomeres II with 8 black-tipped apical teeth, respectively.

***Male genitalia.*** Pygofer (Figs 10–12) wider ventrally than dorsally (approx. 5.8:1), hatchet-shaped in lateral view. Gonostyles (Figs 10, 11) relatively large, broadening towards apex in lateral view (Fig. 10), posterior margin straight, upper margin with dorsally directed, black-tipped process near middle, with ventrally directed, hook-like process near sub-middle on outer upper edge. Anal tube (Figs 10, 12) wide and narrow down in dorsal view, ratio length to width approx. 1.1:1. Aedeagus (Figs 13–15) with one pair of special long endosomal processes, processes with apex acute, sclerotised and pigmented. Phallobase sclerotised and pigmented at base, with two pairs of membranous lobes at apex (Figs 13–15): the dorsal lobes relatively small and the ventral lobes large with complicated ventral lateral lobes in lateral view (Fig. 13), one pair of large lobes in dorsal view (Fig. 15), one pair of large and complicated lobes in ventral view (Fig. 14).

***Female genitalia.*** Segment X (Fig. 17) round and large in dorsal view, ratio length to width at middle approx. 1.3. Gonocoxae VIII with two endogonocoxal processes membranous and ﬂattened on endogonocoxal lobe. Gonopophyses VIII (Fig. 18) sclerotised with six differently sized teeth in lateral view. Gonopophyses IX (Fig. 19) triangular, symmetrical in ventral view, connected at base and separated from 2/3 base. Gonoplacs (Fig. 20) with two sclerotised lobes, ventral lobe with a membranous structure at the top, and lateral lobe with 3-4 long spines at apex.

**Figures 16–20.**
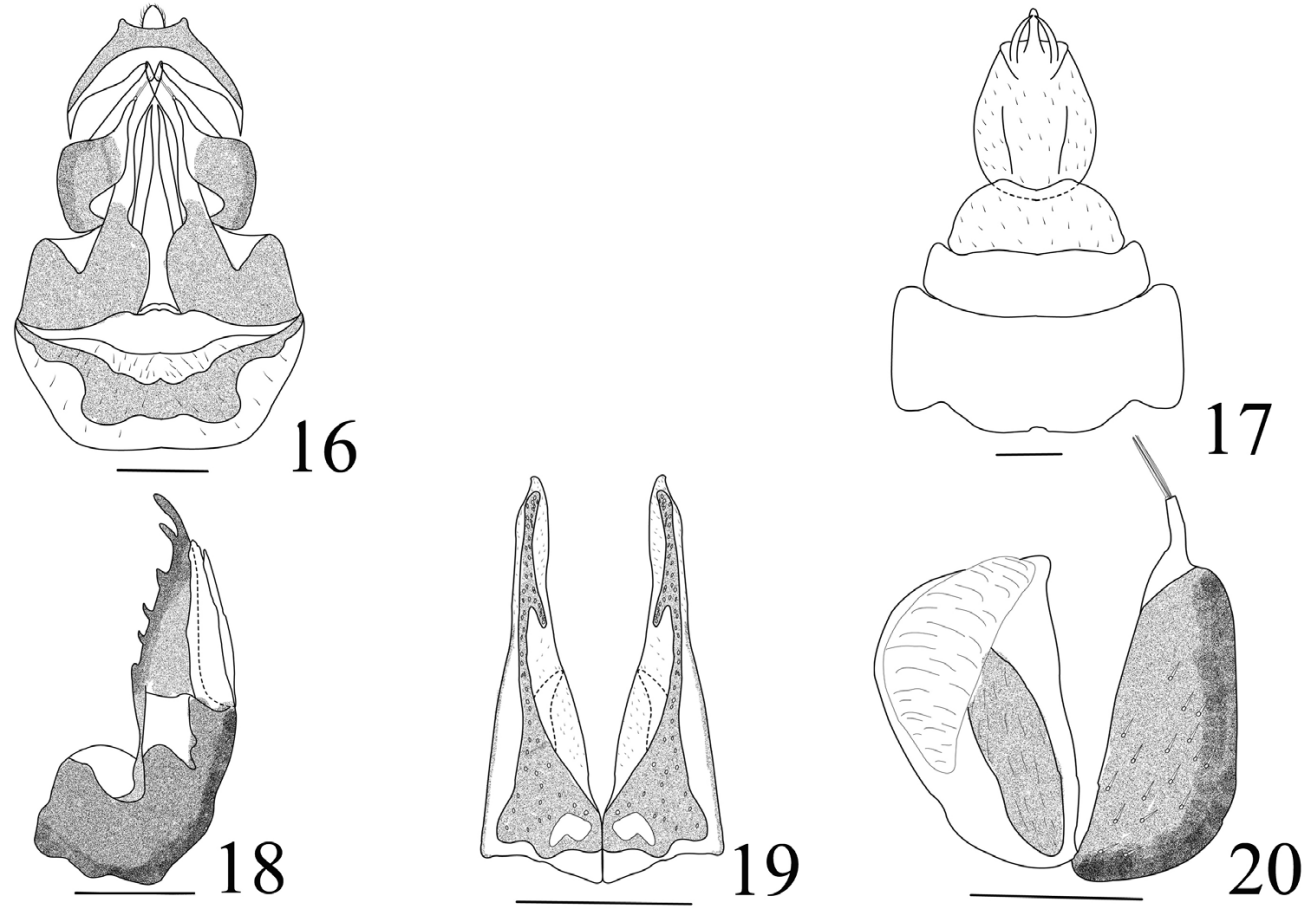
*Miasa dichotoma* sp. n.: **16** genitalia ventral view of female **17** genitalia dorsal view of female **18** gonocoxae VIII (lateral view) **19** gonopophyses IX (ventral view) **20** gonoplacs (lateral view). Scale bars: 0.5 mm.

#### Type material.

Holotype ♂, China, Yunnan, Xishuangbanna, Mengla, 23.VIII.2013, Guo Mei-Na. Paratypes, 1♀, same data as Holotype; 1♂, China, Yunnan, Xishuangbanna, Menglun, 30.VII.2012, Zheng Wei-Bin.

#### Distribution.

China (Yunnan).

#### Differential diagnosis.

This species is similar to *M. trifoliusa* sp. n. but can be distinguished from phallobase. The former has two pairs of membranous lobes of the phallobase at apex, the latter with three pairs of membranous lobes at apex.

#### Etymology.

This new species is named for the Greek word “*dichotoma*” referring to aedeagus that is dichotomous at its apex.

### Miasa trifoliusa

Taxon classificationAnimaliaHemipteraDictyopharidae

Zheng & Chen sp. n.

http://zoobank.org/071117C8-A54B-49A5-8FD7-FB582E751618

Figs 21–32

#### Measurements.

♂, BL: 14.2 mm; HL: 1.8 mm; HW: 0.4 mm; FWL: 10.7 mm.

#### Description.

General colour in dried specimens ferruginous-brown, marked with faint yellow and reddish brown. Cephalic process of the base brown, terminal black, brown on side. Frons brown with faint yellow marks. Frontoclypeal dark with paired brown blotchy markings. Compound eyes dark brown, ocelli light pink. Antennae brown. Pronotum and mesonotum brown, the middle faint yellow. Forewings with stigmal area and posterior margin broadly dull ochreous, a large oblique triangular apical streak, and a narrow streak along nodal line fuscous; hind wings with an apical fuscous spot. Legs brown with dark spot.

**Figures 21–32.**
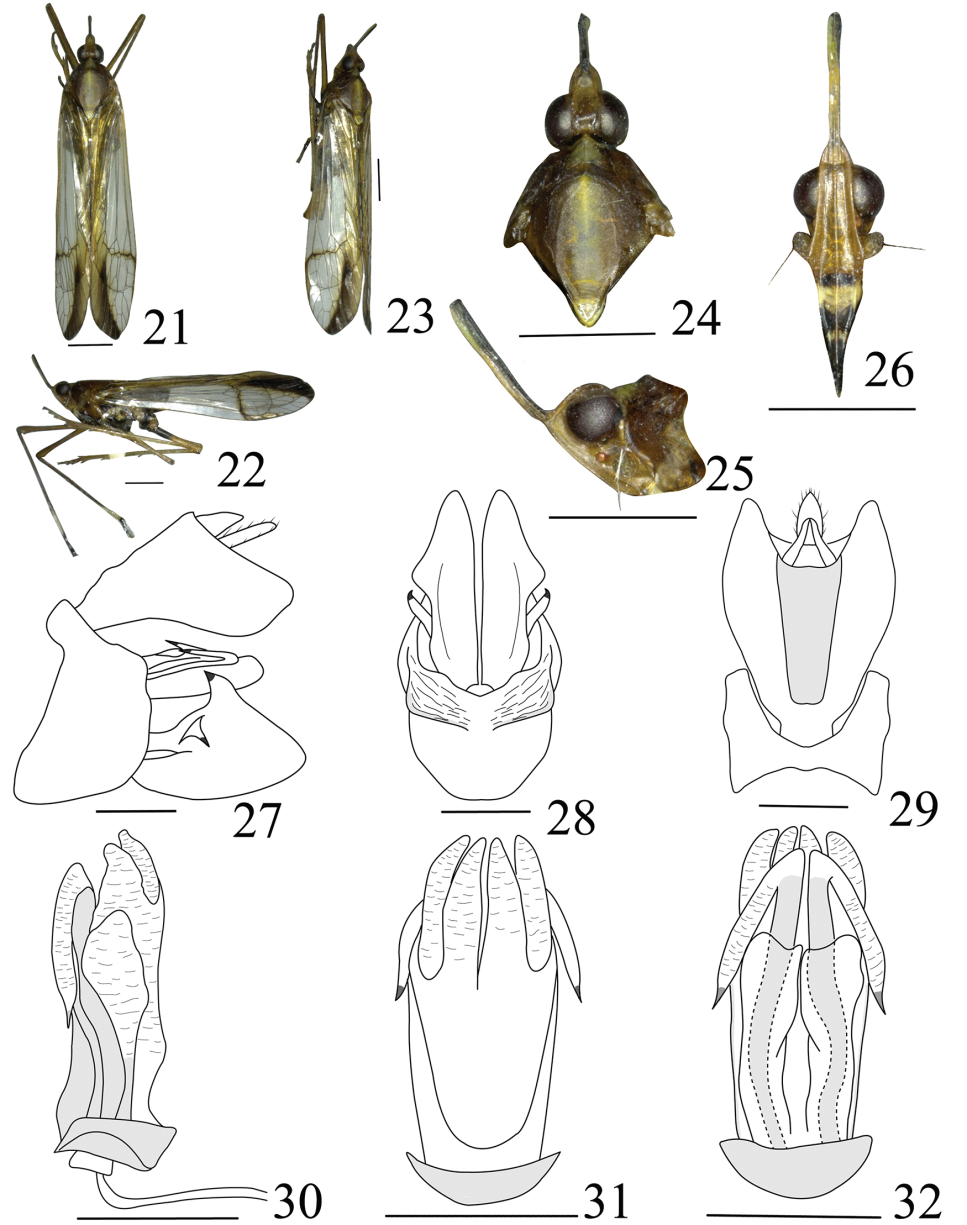
*Miasa trifoliusa* Zheng & Chen, sp. n.: **21** male, holotype, dorsal view **22** male, lateral view **23** male, oblique side view **24** male, head and thorax, dorsal view **25** male, head and pronotum, lateral view **26** male, frons and clypeus, ventral view **27** genitalia, lateral view **28** pygofer and parameres, ventral view **29** pygofer and anal tube, dorsal view **30** aedeagus, lateral view **31** aedeagus, ventral view **32** aedeagus, dorsal view. Scale bars: 2 mm (**21–26**), 0.5 mm (**27–32**).

Cephalic (Figs 21–25) process relatively long, distinct upturned, ratio length to length of pronotum and mesonotum combined 0.7. Vertex (Figs 21–25) with lateral margins carinate, sub-parallel at base, sharply sinuate in front of eyes, then narrowing to arrowhead at apex, ratio of length to width between eyes 3.5. Frons (Fig. 26) elongate, median carina complete and elevated, length approx. 4.8 times long than width. Pronotum (Figs 21–24) distinctly shorter than mesonotum medially in the middle line, median carina obscure, lateral carina distinct, ratio length to length approx. 0.3:1. Mesonotum (Figs 21–24) median carina obscure, lateral carina distinct. Forewings (Figs 22, 23) elongate, with ratio of length to width approx. 4.0:1; stigma distinct, with four cells, CuA vein first branched before Sc+R and M veins near middle; crossveins very scarce, forming a nodal line along Sc+R, M and CuA veins at apical 1/3; apical cells approx. 12; Pcu and A_1_ veins fused into a long Pcu+A_1_ vein at apical 1/6 in clavus. Legs long and thin, fore femur not flattened and dilated, with one minute, short, blunt spine near apex; hind tibia with five lateral black-tipped spines and six apical black-tipped teeth, hind tibiae I with ten and tarsomeres II with eight black-tipped apical teeth, respectively.

**Male genitalia.** Pygofer (Figs 27–29) wider ventrally than dorsally (aprpox. 4.5:1), hatchet-shaped in lateral view. Gonostyles (Figs 27, 28) relatively large, broadening towards apex in lateral view (Fig. 27), posterior margin straight, upper margin with dorsally directed, black-tipped process near middle, with ventrally directed, hook-like process near sub-middle on outer upper edge. Anal tube (Figs 27, 29) wide and narrow down in dorsal view, ratio length to width approx. 1.4:1. Aedeagus (Figs 30–32) with one pair of special long endosomal processes, processes with apex acute, sclerotised and pigmented. Phallobase sclerotised and pigmented at base, with three pairs of membranous lobes at apex: the dorsal lobes relatively small and the ventral two pairs of membranous lobes large and connected in ventral view (Fig. 31).

#### Type material.

Holotype ♂, China, Yunnan, Xishuangbanna Menglun. 18.VIII.2014, Wang Ying-Jian.

#### Distribution.

China (Yunnan).

#### Differential diagnosis.

This species is similar to *M. wallacei* Muir, but can be distinguished most easily by the phallobase conformation. The former is membranous with a pair of dorsal lobes directed posteriorly, and two pairs of ventral lobes: large, almost equal in length, apically bifurcate, directed dorsally, the latter membranous with a pair of dorsal lobes directed posteriorly, and two pairs of ventral lobes: upper pair large and elongate, directed dorsally; lower pair relatively small and rounded. There have differences in body colour and in the lengths and widths of the forewings, but the differences are not obvious.

#### Etymology.

This new species is named with the Greek word “*trifoliusa*” referring to phallobase with three pairs of membranous lobes at apex.

## Discussion

The discovery of these two new species broadens our knowledge of the morphology and biogeography of the genus, although it is not a new record of the genus for China. The two new species occur in Yunnan, China (as does *M. wallacei*). This might be related with the special geographical position and climate of Yunnan.Tto the northwest lies Lancang County; to the southeast, south, and southwest respectively there are borders with Laos, Burma, and Vietnam. These regions and countries are linked by mountains and rivers and are located on the Tropic of Cancer in tropical humid areas.

## Supplementary Material

XML Treatment for Miasa

XML Treatment for Miasa dichotoma

XML Treatment for Miasa trifoliusa
